# Improving Selection Efficiency of Crop Breeding With Genomic Prediction Aided Sparse Phenotyping

**DOI:** 10.3389/fpls.2021.735285

**Published:** 2021-10-06

**Authors:** Sang He, Yong Jiang, Rebecca Thistlethwaite, Matthew J. Hayden, Richard Trethowan, Hans D. Daetwyler

**Affiliations:** ^1^Agriculture Victoria, AgriBio, Centre for AgriBioscience, Bundoora, VIC, Australia; ^2^CAAS-IRRI Joint Laboratory for Genomics-Assisted Germplasm Enhancement, Agricultural Genomics Institute in Shenzhen, Chinese Academy of Agricultural Sciences, Shenzhen, China; ^3^Department of Breeding Research, Leibniz Institute of Plant Genetics and Crop Plant Research (IPK), Gatersleben, Germany; ^4^School of Life and Environmental Sciences, Plant Breeding Institute, Sydney Institute of Agriculture, The University of Sydney, Narrabri, NSW, Australia; ^5^School of Applied Systems Biology, La Trobe University, Bundoora, VIC, Australia; ^6^School of Life and Environmental Sciences, Plant Breeding Institute, Sydney Institute of Agriculture, The University of Sydney, Cobbitty, NSW, Australia

**Keywords:** sparse phenotyping, genomic prediction, multi-environment trials, response to selection, correlations between environments

## Abstract

Increasing the number of environments for phenotyping of crop lines in earlier stages of breeding programs can improve selection accuracy. However, this is often not feasible due to cost. In our study, we investigated a sparse phenotyping method that does not test all entries in all environments, but instead capitalizes on genomic prediction to predict missing phenotypes in additional environments without extra phenotyping expenditure. The breeders’ main interest – response to selection – was directly simulated to evaluate the effectiveness of the sparse genomic phenotyping method in a wheat and a rice data set. Whether sparse phenotyping resulted in more selection response depended on the correlations of phenotypes between environments. The sparse phenotyping method consistently showed statistically significant higher responses to selection, compared to complete phenotyping, when the majority of completely phenotyped environments were negatively (wheat) or lowly positively (rice) correlated and any extension environment was highly positively correlated with any of the completely phenotyped environments. When all environments were positively correlated (wheat) or any highly positively correlated environments existed (wheat and rice), sparse phenotyping did not improved response. Our results indicate that genomics-based sparse phenotyping can improve selection response in the middle stages of crop breeding programs.

## Introduction

Genomic selection is a promising tool to assist plant breeding by accelerating selection gain per unit time (Endelman et al., 2014; Slater et al., 2016; Crossa et al., 2017; Voss-Fels et al., 2019). In crop breeding programs, there is a consensus that genomic selection should be applied in the early stages as phenotyping intensity during this period is low, especially for grain yield and hard-to-measure traits (Endelman et al., 2014; He et al., 2016). However, this genomic selection strategy depends on an independent and robust reference population, normally consisting of historical data collected across several years (Dawson et al., 2013; Rutkoski et al., 2015; Jarquin et al., 2016).

Another way to deploy genomic selection in breeding is through phenotype imputation (Hori et al., 2016), which does not require an independent reference population. In the middle stages of breeding programs (e.g., sometimes referred to as stages one or two), crop lines are regularly phenotyped in only a few environments. Increasing the number of testing environments during these stages with genomic selection could markedly boost selection accuracy, compared to the advanced stages where most selection candidates are intensively tested in many environments (He et al., 2016). However, budget and seed availability constraints make complete phenotyping of all selection candidates in many environments impractical earlier in the breeding program. Nevertheless, the phenotype imputation scheme proposed by Hori et al. (2016) suggests that lines do not need to be tested in each environment, i.e., sparse phenotyping. Instead, the phenotype of lines in untested environments is reliably predicted using methods such as multi-environment genomic prediction approaches based on the remaining observations in tested environments. Consequently, a multi-environment trial (MET) with more testing environments could improve overall selection accuracy.

Traditionally, the correlation between the best linear unbiased estimation (BLUE) of genetic value and the genomic estimated genetic value (GEGV) is used to evaluate genomic prediction accuracy (Heslot et al., 2012; Rutkoski et al., 2015; He et al., 2016; Jarquin et al., 2016). BLUEs are assumed to be the best benchmark of GEGV because they are derived directly from *per se* performance, which is trusted by plant breeders. However, the true genetic value is unknown and whether BLUE or GEGV is closer to the true genetic value is difficult to establish. Thus, rather than prediction accuracy, the focus could be on the actual breeders’ interest, e.g., the response to selection, which can be inferred from a simulation-based approach (Piepho and Möhring, 2007) to directly evaluate the effectiveness of genomic selection. To our knowledge, no study has applied this approach to assess the effectiveness of genomic selection.

Our study utilized an Australian pre-breeding wheat population and a publicly available rice pureline population, both with complete and orthogonal phenotypic records of grain yield across 3years and two sowing times, to investigate the potential of genomics-assisted sparse phenotyping to improve selection response in the context of multi-environment trials. We also investigate the relationship among environments and how this affects the effectiveness of the proposed genomics-assisted sparse phenotyping method.

## Materials and Methods

### Wheat Data Set

The wheat grain yield data set used in this study originated from the data set used in He et al. (2019), which consisted of five individual data sets including 1,351 genotypes. The genotypes were evaluated from year 2012 to 2017 with two times of sowing (TOS) per year at Narrabri in north-western New South Wales, Australia. The randomized complete block design with two replicates was applied to measure five agronomic traits incl. Grain yield, plant height, protein content, screenings percentage, and thousand kernel weight. The experiments in the current study were based on 189 lines consistently tested from year 2015 to 2017 at two TOS per year. These lines composed an orthogonal data set with a dimension of 189 lines and six environments.

Phenotypic analysis was implemented for each data set to derive the repeatability estimate per environment (year–TOS combination) and best linear unbiased estimates (BLUEs) per line in each environment, as described in He et al. (2019). Specifically, the phenotypic data of each environment were analyzed using a mixed linear model. The field design relevant effects such as range, row, and replicates as well as residual effect were all designated as random effects which followed an identical and independent normal distribution. Genetic effects were in tandem treated as fix and random to derive the best linear unbiased estimates (BLUE) and repeatability of each environment. Another mixed linear model based on BLUE of the 189 genotypes in each environment was fitted to estimate the heritability of grain yield, which was formulated as $\hat{y}=1_{n}\mu +Z_{r}r+Z_{l}l+\varepsilon ,$ where n is the number of BLUE values, $\hat{y}$ is n-dimensional vector of genotype BLUEs across environments, μ is the common intercept, **1**_**n**_ is a n-dimensional vector of ones, **r** is the vector of environment effects, **l** is the vector of genetic effects of genotypes, **Z**_**r**_ and **Z**_**l**_ are incidence matrices for **r** and **l**, and **ε** is the random residual. Effects **r**, **l**, and **ε** were fitted as random effects following identical and independent normal distributions. The heritability of grain yield was estimated using formula: $1-\frac{\bar{c}}{2\sigma_{l}^{2}}$, where $\bar{c}$ is the mean variance of a difference between two best linear unbiased predictions (BLUP) of genetic effects of genotypes (Cullis et al., 2006).

The genotypic data of the 189 lines used in this study were drawn from the genotypic data of 1,351 wheat lines fingerprinted with 41,666 90K single nucleotide polymorphisms (SNP) in He et al. (2019). As the number of genotypes was reduced, SNPs were refiltered by removing those with a minor allele frequency of less than 0.05, which left 32,800 SNP for subsequent analyses. The genetic diversity of the 189 genotypes was inspected based on a cluster analysis using Rogers’ distance (Roger, 1972) estimated by the 32,800 SNP. The correlation between environments was estimated by Pearson correlation coefficient between the BLUEs of the 189 genotypes in different environments.

### Rice Data Set

The publicly available rice data set (Spindel et al., 2015) included 358 rice lines phenotyped for six agronomic traits across 4years and two seasons, i.e., eight environments (year–season combinations). As in wheat, phenotypic analyses included estimation of repeatability per environment and BLUEs per line. Based on the BLUEs, we selected six environments with the greatest range in correlations between environments out of the total eight environments to evaluate the effectiveness of the sparse phenotyping method. Finally, 160 lines were available with orthogonal yield phenotypic data in all six environments. Genotyping-by-sequencing (GBS) genotypes for 108,024 SNPs were quality controlled as follows. Low-quality SNPs with MAF less than 0.05 and call rate less than 0.9 were removed. Eventually, 46,232 SNPs were available for the 160 used lines. The correlation between environments was estimated by Pearson correlation coefficient between the BLUEs of the 160 genotypic lines in different environments.

### Multi-Environment Genomic Prediction Model

A multi-environment genomic prediction model explicitly describing genotype-by-environment interactions was used:


$$y=1_{mn}\mu +Z_{v}v+Z_{g}g+gv+e$$

where *m* is the number of environments, *n* is the number of genotypes, **y** is a *m*×*n* vector of BLUEs of genotypes in each environment, μ is the common intercept, **v** is the *m*-dimensional vector of environment main effect, **g** is the *n*-dimensional vector of additive genetic main effect of genotypes, **gv** is the *m*×*n* vector of genotype-by-environment interaction effects, **e** is the random residual, **Z**_**v**_ is the incidence matrices for **v**, and **Z**_**g**_ is the incidence matrices for **g**. We assumed $v\sim N\left(0,I\sigma_{v}^{2}\right)$, $g\sim N\left(0,G\sigma_{g}^{2}\right)$, $gv\sim N\left(0,\left[Z_{g}GZ_{g}^{\prime }\right]⊙Z_{v}Z_{v}^{\prime }\sigma_{\mathrm{gv}}^{2}\right)$, and $e\sim N\left(0,I\sigma_{e}^{2}\right)$, where ⊙ is the Hadamard product of matrices, $\sigma_{g}^{2}$, $\sigma_{\mathrm{gv}}^{2},$ and $\sigma_{e}^{2}\;$are their variance components, respectively, for genotype, genotype-by-environment interaction effects, and random residual. **G** is the genomic relationship matrix proposed by VanRaden (2008) constructed based on SNP genotypic profiles. The genomic prediction model was run in R (R Core Team, 2016) using the BGLR package (de los Campos and Pérez-Rodríguez, 2016). Iteration times were fixed to 30,000, and the first 5,000 times were set as burn-in.

### Sparse Phenotyping Method

We compared the selection response of the complete phenotyping trial in fewer environments with a sparse genomic phenotyping method in additional environments. In this sense, all possible combinations of three environments out of the total six environments were used as the complete phenotyping trials, which retained total phenotypic values (BLUEs per environment). Phenotypic values in combinations of four, five, and six environments (there is just one combination using all six environments) were proportionally masked to create the sparse phenotyping trials. The percentage of phenotypic values retained in the 4-, 5-, and 6-environment combinations was 75, 60, and 50%, respectively, which made the phenotyping intensity in all 3-, 4-, 5-, and 6-environment combinations equivalent. Thus, the number of BLUEs and the amount of phenotype data collected was the same in all scenarios. There were 20 different combinations of three environments out of the total six environments. Each 3-environment combination was extended to three 4- or 5-environment combinations by including one or two environments from the remaining three environments. According to the phenotyping proportions (75, 60, and 50%) of 4-, 5-, and 6-environment combinations, phenotypic values in each 4-, 5-, and 6-environment combination were randomly masked one hundred times according to the cross-validation strategy two (CV2) in He et al. (2019). Specifically in this study, each genotype has six environment-specific BLUEs. We first attempted to randomly mask one BLUE of genotypes in the 4-, 5-, and 6-environment combinations to make the phenotyping proportions the same as the 3-environment complete phenotyping trial. If masking one BLUE was insufficient to meet the required phenotyping proportion, another BLUE of genotypes was masked until the required phenotyping proportion was reached.

### Response to Selection

The genomic prediction model, also known as a mixed linear model, can be used to directly estimate the response to selection through a simulation-based approach following Piepho and Möhring (2007). Briefly, the multi-environment genomic prediction model was fitted using phenotypic records of complete phenotyping trial (3-environment combination) and phenotypic records of sparse phenotyping trials (4-, 5-, and 6-environment combinations). We were mainly interested in the relationship between the true genetic main effect **g** and its best linear unbiased prediction (BLUP) $\hat{g}$, because the selection was based on the BLUP, while the response of selection was determined by the true values. In fact, the joint distribution of **g** and $\hat{g}$ is multivariate normal and the corresponding variance–covariance matrix Ω=vargg^ can be derived from the mixed model equations. Then, Ω was eigendecomposed as $\Omega =D\Lambda D^{\prime }=\Gamma \Gamma^{\prime }$, where **D** is the matrix of eigenvectors and **Λ** is the diagonal matrix of eigenvalues, $\Gamma =D\sqrt{\Lambda }$. The vector combining the true and predicted genetic main effects w=gg^ could be simulated by w=Γz, where **z** is a 2n-dimensional vector of independent standard normal deviates because $\mathrm{var}\left(w\right)=\mathrm{var}\left(\Gamma z\right)=\Gamma \mathrm{var}\left(z\right)\Gamma^{\prime }=\Gamma \Gamma^{\prime }=\Omega$ as desired.

For each 3-environment complete phenotyping trial, the responses to selection under varying selection ratios (corresponding to different selection intensities) ranging from 10 to 90% with a gap of 10% were simulated 10,000 times. In each simulation run, the vector **w** combining the true and predicted genetic main effects was simulated and a subset of genotypes (S_q_) with top p% (*p*=10–90) of $\hat{g}$ was selected. The response to selection of the simulation run (q^th^) was calculated as $R_{q}=\frac{\sum_{i\in S_{q}}g_{i}}{\#\left(S_{q}\right)}$, where $\#\left(S_{q}\right)$ is the size of S_q_. For each selection ratio (10–90%), the average value of response to selection of the 10,000 runs was finally used as the achieved responses to selections of the complete phenotyping trial, i.e., $R=\frac{\sum_{q=1}^{10000}R_{q}}{10000}$. The responses to selections of each extended 4-, 5-, and 6-environment sparse genomic phenotyping trial scenario were simulated in the same manner based on only unmasked phenotypic values. The effectiveness of genomic selection was determined by comparing the achieved selection response between each complete phenotyping trial and its extended different sparse phenotyping trials. The difference between the achieved response of the complete phenotyping scenarios and responses from one hundred replicates of the corresponding extended sparse phenotyping scenarios (with random phenotype masking) under each selection ratio (10–90%) was statistically tested with Student’s *t* tests.

## Results

### Phenotypic Data and Population Structure

**Figure 1:**
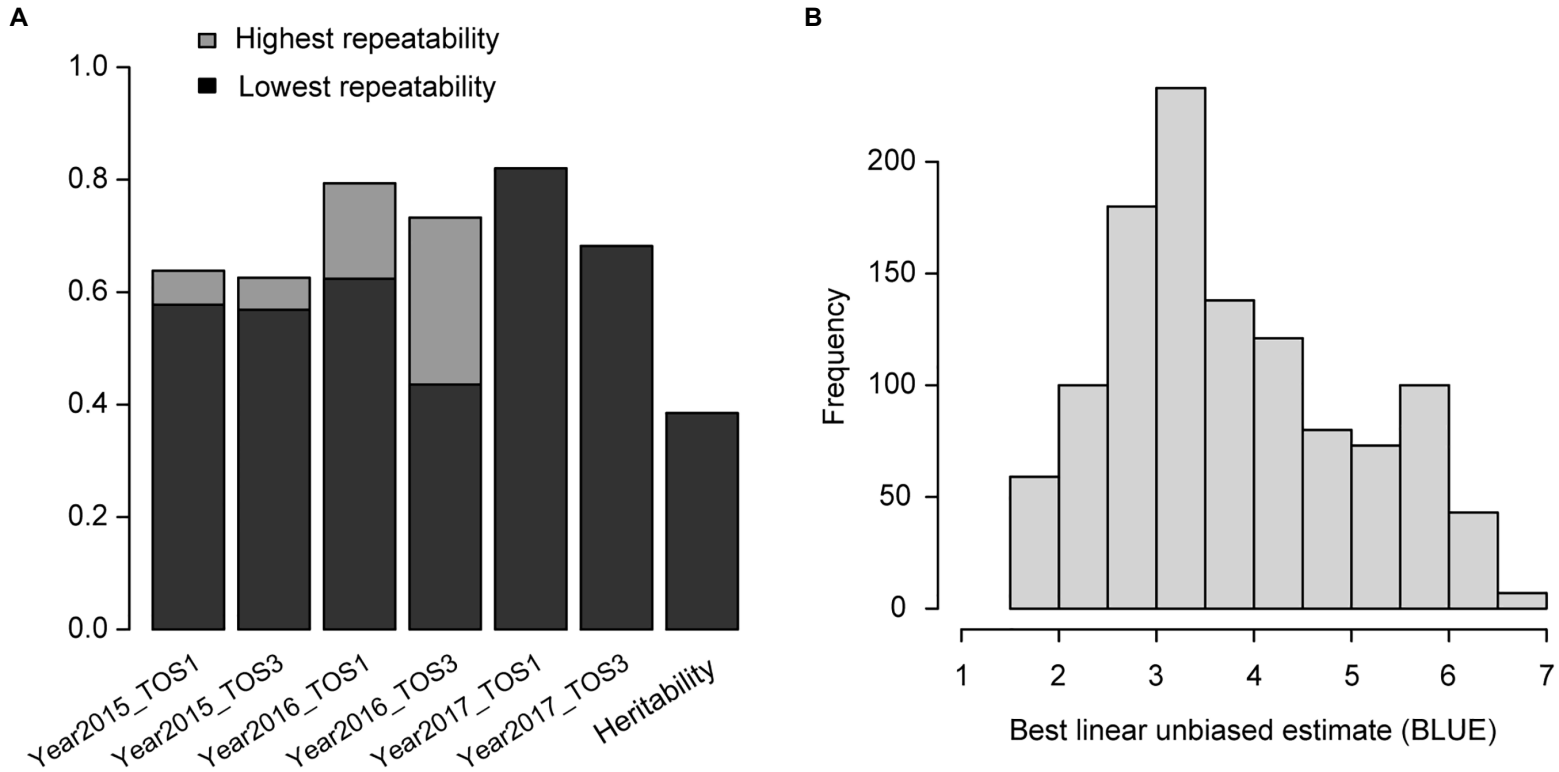
Wheat – **(A)** heritability of grain yield and repeatability in each environment. The highest and lowest repeatability of specific environments evaluated in different data sets are shown in two grayscales; **(B)** distribution of best linear unbiased estimate (BLUE) of genotypes in different environments.

For the wheat data set, the overall heritability of grain yield was 0.38 and repeatability of each environment was above 0.4, indicating that the phenotypic data were of high quality (Figure 1A). The distribution of BLUEs in different environments was asymptotically normal (Figure 1B). Several large families were identified by clustering analysis and linkages existed across families (Supplementary Figure 1). The Rogers’ distance values between any pair of genotypes ranged from 0.01 to 0.53. For the rice data set, the overall heritability was 0.83 and repeatability of each environment was over 0.4 (Supplementary Figure 2A). The distribution of BLUEs across different environments was near normal (Supplementary Figure 2B).

### Correlations Between Environments

**Figure 2:**
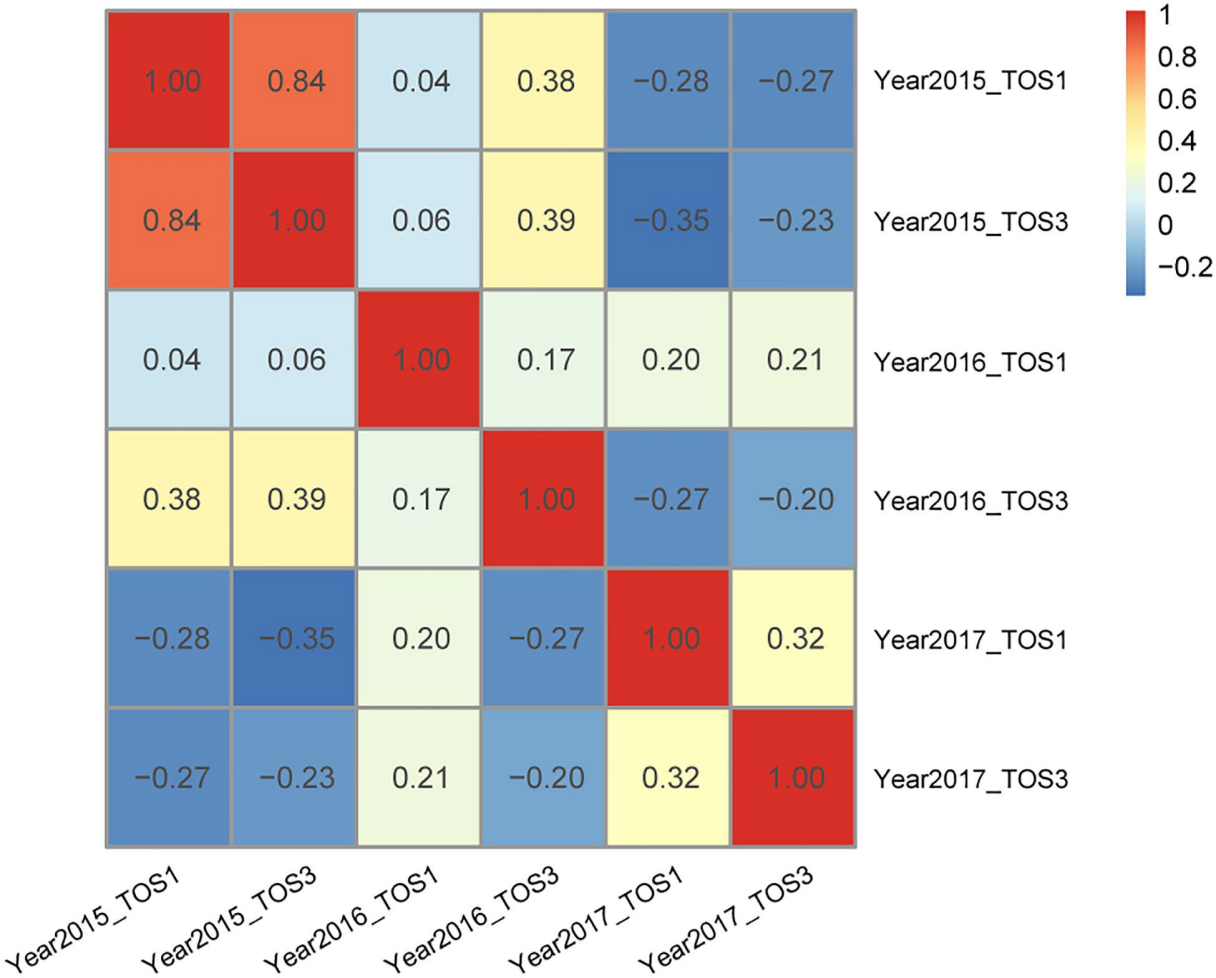
Wheat – pairwise correlation between environments.

In the wheat data set, pairwise correlations ranged from −0.35 to 0.84 among the six environments (Figure 2). Among the 3-environment combinations, five combinations showed all positive pairwise correlations. Each 3-environment combination displayed at least one positive pairwise correlation (Supplementary Table 1). Inspecting the pairwise correlations within the twenty 3-environment combinations, four groupings became clear: (1) one pair of environments had high positive correlation 0.84, i.e., combinations 1–4; (2) environments where all pairwise correlations were positive, i.e., combinations 5, 11, and 19; (3) one pair of environments had negative correlations, i.e., combinations 6–7, 12–13, and 17–18; and (4) two pairs of environments had negative correlations, i.e., combinations 8–10, 14–16, and 20 (Supplementary Table 1).

In the rice data set, correlations of pairs of environments varied from 0.05 to 0.67 (Supplementary Figure 3). Among the 3-environment combinations, in one combination all correlations were below 0.18 and four combinations had one highly positive correlation of 0.67 (Supplementary Figure 3). Based on the pairwise correlations within the twenty 3-environment combinations, there were four distinct groupings: (1) one pair of environments with high positive correlation 0.67, i.e., combinations 10, 16, 19, 20; (2) all pairwise correlations moderately positive above 0.18, i.e., combinations 12, 13, 17, 18; (3) one pair of environments lowly positively correlated below 0.18, i.e., combinations 3, 4, 6–9, 11, 14, 15; and (4) more than one pair of environments lowly positively correlated below 0.18, i.e., 1, 2, 5 (Supplementary Figure 3).

### Simulated Response to Selection

**Figure 3:**
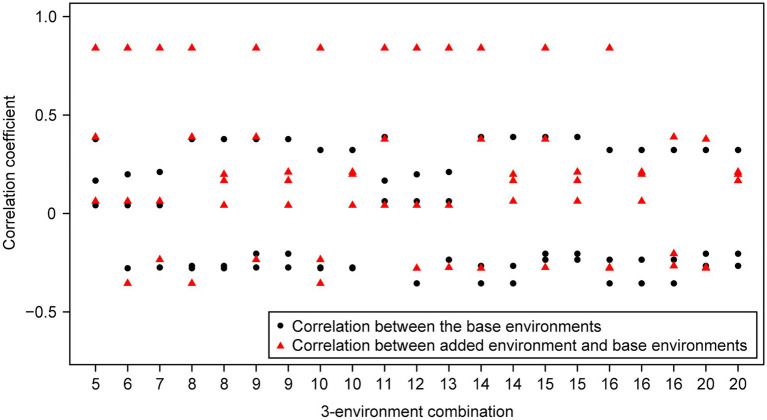
Wheat – 3-environment combinations with complete phenotypic values showing statistically significant (*p*<0.05) lower response to selection than their extended 4-environment combinations using genomics-assisted sparse phenotyping. Labels of horizontal axis are the scenario numbers of 3-environment combinations. Black dots represent correlation coefficients between the three base environments with complete phenotypic values. Red triangles indicate correlation coefficients between the added environment and base environments.

**Figure 4:**
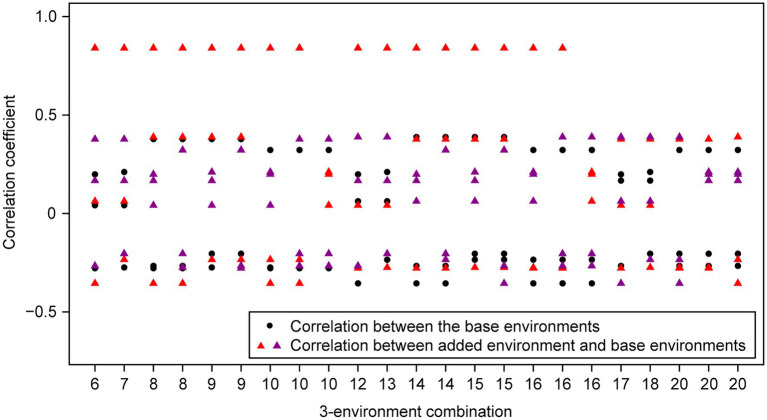
Wheat – 3-environment combinations with complete phenotypic values showing statistically significant (*p*<0.05) lower response to selection than their extended 5-environment combinations using genomics-assisted sparse phenotyping. Labels of horizontal axis are the scenario numbers of 3-environment combinations. Black dots represent correlation coefficients between the three base environments with complete phenotypic values. Triangles with different colors indicate correlation coefficients between separate added environments, i.e., the first or second added environment, and base environments.

**Figure 5:**
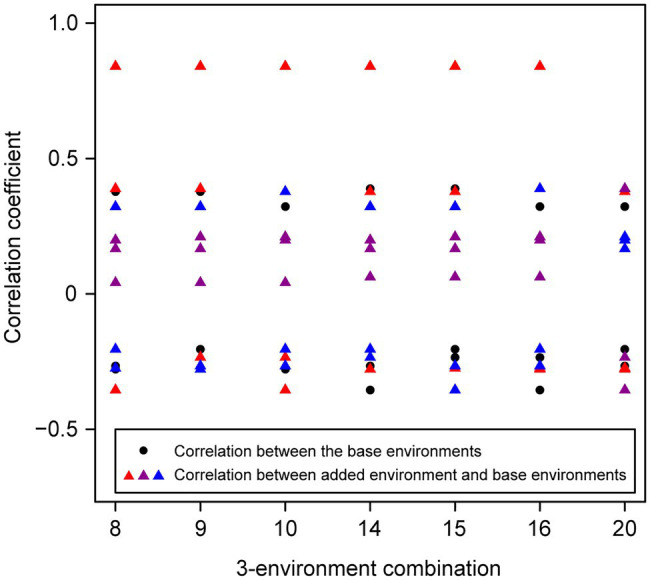
Wheat – 3-environment combinations with complete phenotypic values showing statistically significant (*p*<0.05) lower response to selection than using total six environments with genomics-assisted sparse phenotyping. Labels of horizontal axis are the scenario numbers of 3-environment combinations. Black dots represent correlation coefficients between the three base environments with complete phenotypic values. Triangles with different colors indicate correlation coefficients between separate added environments, i.e., the first, second, or third added environment, and base environments.

**Figure 6:**
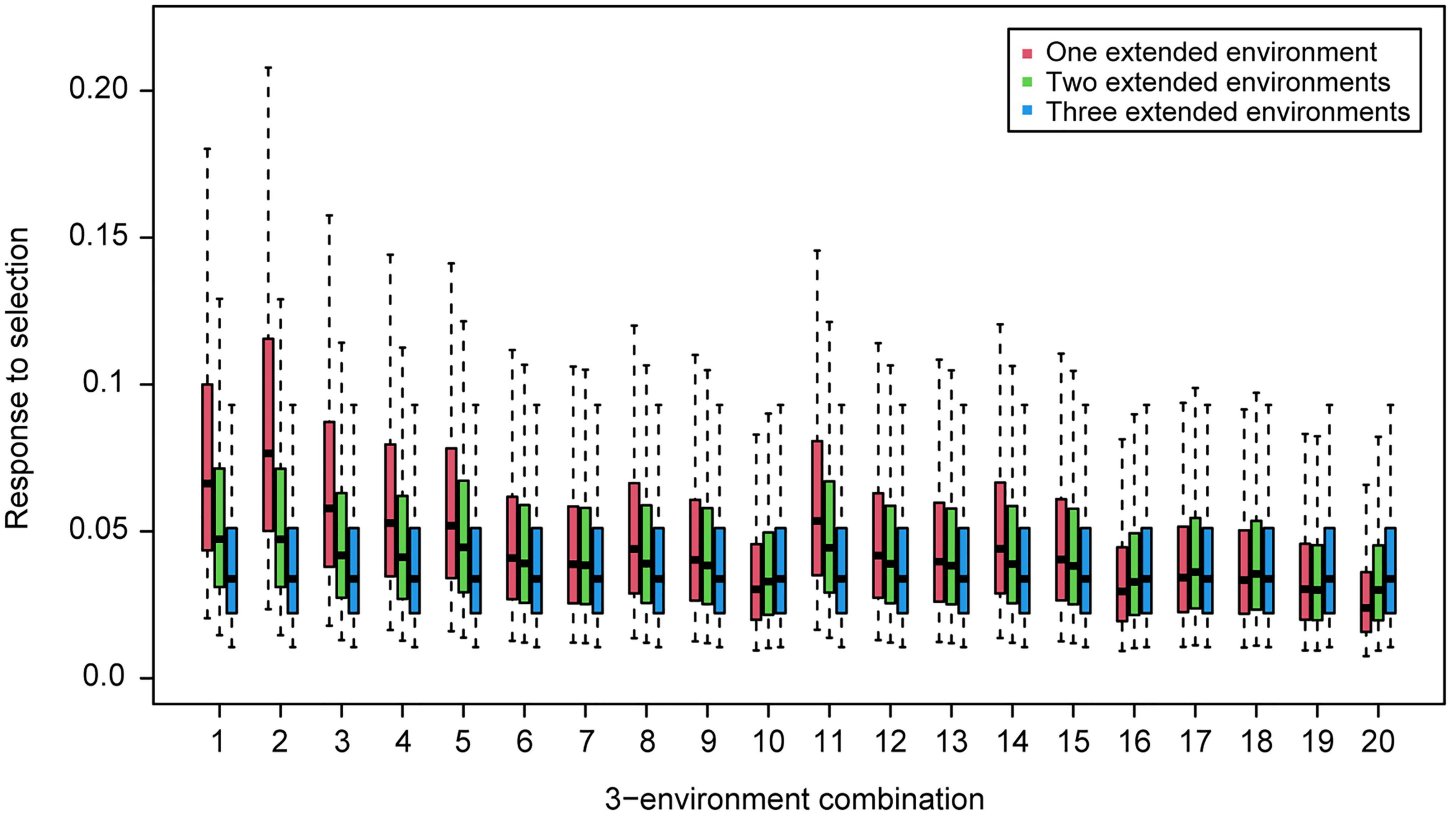
Wheat – responses to selection of 4-environment (one extended environment), 5-environment (two extended environments) and 6-environment (three extended environments) sparse phenotyping combinations belonging to each 3-environment complete phenotyping combination. Labels of horizontal axis are the scenario numbers of 3-environment combinations.

For the wheat data set, twenty-one 4-environment combinations with sparse phenotyping applied had statistically significant higher responses to selection, compared to their equivalent 3-environment combination with complete phenotyping under each selection ratio, i.e., 10–90% (Figure 3). Most of the combinations contained one negative correlation between the three base environments with complete phenotypic records and one highly positive correlation (0.84) between the extension environment and the base environments (Figure 3). For the 5- and 6-environment combinations, there were twenty-three and seven sparse combinations showing higher response, respectively (Figures 4, 5). One negative correlation between the base environments and one highly positive correlation between expansion environment and base environments were also observed in the 5- and 6-environment combinations (Figures 4, 5). Comparison of the responses of all 3-environment combinations and their extended 4-, 5-, and 6-environment combinations identified five 3-environment combinations where the sparse phenotyping combinations did not result in a significantly higher response than the corresponding full 3-environment scenarios (combinations 1–4, 19; Supplementary Table 2). For most 3-environment complete phenotyping combinations, the responses achieved by the extended 4-environment sparse phenotyping scenarios were the highest compared to the 5- and 6-environment combinations (Figure 6).

For the rice data set, twenty-five 4-environment combinations sparse phenotyping scenarios showed statistically significant higher responses to selection than their corresponding 3-environment complete phenotyping combination under each selection ratio, i.e., 10–90% (Supplementary Figure 4). Most of these included two lowly positive correlations (<0.18) within the three complete phenotyping environments and/or one highly positive correlation (0.67) between the extended environment and one complete phenotyping environment (Supplementary Figure 4). For the 5- and 6-environment combinations, there were twenty-one and seven combinations, respectively, displaying higher response (Supplementary Figures 5, 6). Again, one highly positive correlation between the expansion environment and base environments and at least two lowly positive correlations within the base environments were observed in the 5- and 6-environment combinations (Supplementary Figures 5, 6). The 3-environment combinations with one highly positive correlation, i.e., group 1, showed no improved response from sparse phenotyping (Supplementary Figures 4–6). The responses of 4-environment sparse combination with one extended environment tended to be higher than those of 5- and 6-environment sparse combinations (Supplementary Figure 7).

## Discussion

Our study investigated the potential of a genomics-assisted sparse phenotyping method *via* simulated selection responses based on a wheat and a rice data set. Results of both data sets showed that the sparse phenotyping can lead to a similar or greater response and provides information on genotype performance in more environments, compared to fully replicated trials. As the level of phenotyping (i.e., the number of observations) was the same in all scenarios, the advantage of sparse phenotyping was achieved with a similar budget. While families existed in the populations, our sparse phenotyping method tested each genotype in at least one environment. Consequently, as all genotypes were included in the reference set, the families did not introduce bias due to relatedness discrepancy to genomic prediction in the different phenotype masking scenarios.

### Inclusion of Environment Correlation in Genomic Prediction Model Reduces the Benefit of Genomics-Assisted Sparse Phenotyping

In our study, a basic multi-environment genomic prediction model considering environments independent was used to simulate response to selection. Nevertheless, a sophisticated model that accommodates correlation between environments seems more reasonable in theory and more suited to be implemented. Jarquin et al. (2014) and Saint Pierre et al. (2016) demonstrated using environmental descriptors such as weather data to describe environmental relationship could improve genomic prediction accuracy. However, such environmental data are not always available. Martini et al. (2020) proposed to straightforwardly use phenotypic correlation of overlapped genotypes in different environments to specify the environmental relationship matrix. Thus, we also tested the effectiveness of the model in the wheat data set using correlation between BLUEs of unmasked genotypes in both environments to compile the environmental relationship matrix. Results showed that the sophisticated model including environmental correlation reduced the number of cases where the sparse phenotyping method displayed significantly higher response than complete phenotyping, as compared to the basic model (Supplementary Figures 8–10). This may be attributed to the number of genotypes used in our study being insufficient to reliably estimate the environmental relationship matrix (Martini et al., 2020). For the sparse phenotyping scenarios, the number of genotypes that can be used to estimate environmental relationship matrix, i.e., unmasked genotypes in both environments, would decrease even more. Particularly, when a total six environments were used, there was only one combination in which the sparse phenotyping performed significantly better (Supplementary Figure 10). This is because when the number of expansion environments increased, the number of unmasked genotypes with phenotypes in all environments reduced (Supplementary Figure 11), leading to a reduction in the reliability of correlation estimates. Alternatively, a more sophisticated model with unstructured environment covariances was also fitted (Burgueño et al., 2012). However, the phenotypic variance–covariance matrix was not always invertible when the sparse phenotyping pattern changed. Based on these results, we recommend to use the basic multi-environment genomic prediction model to compare the effectiveness of sparse and complete phenotyping strategies unless there are adequate common genotypes in different environments available to reliably estimate the environmental relationship matrix.

### Effectiveness of Sparse Phenotyping Could Be Further Improved by Selective Phenotyping

Our study used a simple stochastic masking design to simulate the sparse phenotyping patterns on the basis that each genotype was tested in at least one environment. However, a more sophisticated selective phenotyping design could help improve the effectiveness of sparse phenotyping (Heslot and Feoktistov, 2020; Jarquin et al., 2020). Jarquin et al. (2020) proposed to completely phenotype a small proportion of genotypes in all environments to facilitate the estimation of environmental variance. As a result, substantial savings of phenotyping cost can be achieved while a high prediction accuracy was maintained. Heslot and Feoktistov (2020) demonstrated that precisely selecting a subset of genotypes for phenotyping based on relatedness could optimize the estimation of marker effect and tremendously increase prediction accuracy compared to randomly selecting a subset with equal size. This suggests that the unit of selection could shift to alleles being sufficiently replicated across environments. Therefore, instead of phenotyping each line in at least one environment, selecting a subset of lines would capitalize on genetic relationship and adding emphasis by testing some individuals in more environments to boost the overall phenotyping intensity could in turn further improve the effectiveness of sparse phenotyping. In this sense, further studies are needed to substantiate the merit of selective phenotyping design on promoting simulated response to selection of sparse phenotyping.

### The Benefit of Sparse Phenotyping Can Be Anticipated From Correlations Between Environments

The correlations between environments in the wheat data set included high (e.g., 0.84), moderate (e.g., 0.32 and 0.38), low (e.g., 0.04 and 0.06), and negative (e.g., −0.28 and −0.35), which is representative of the types of environments encountered in plant breeding. These four groupings of 3-environment combinations are illustrated in Table 1 and can be used to understand when sparse phenotyping can be beneficial.

**Table 1:** Wheat – grouping of 3-environment combinations according to their utility of genomics-assisted sparse phenotyping over complete phenotyping.

Group	Complete phenotyping group correlation characteristics	Complete phenotyping three-environment combinations	Genomic sparse phenotyping better?		
			Plus 1 sparse environment	Plus 2 sparse environments	Plus 3 sparse environments
1	One highly positive correlation	1, 2, 3, 4	No	No	No
2	All correlations positive	5, 11, 19	Yes, when additional environment was positively highly correlated with the complete phenotyping environment	No	No
3	One negative correlation	6, 7, 12, 13, 17, 18	Yes, when additional environment was positively highly correlated with the complete phenotyping environment	Yes, when additional environments were positively highly or moderately correlated with the complete phenotyping environment, where the two moderately correlated environments need to be highly correlated	No
4	Two negative correlations	8, 9, 10, 14, 15, 16, 20	Yes, when additional environment was positively highly correlated with any or positively correlated with all complete phenotyping environments	Yes, when one additional environment was positively highly correlated with the complete phenotyping environment	Yes, when one additional environment was positively highly correlated with the complete phenotyping environment

Group 1 had a highly positive correlation (0.84) between environments and the sparse phenotyping method did not result in additional selection response, regardless of the number of expansion environments added (Table 1; Figures 3–5).

In group 2, all pairwise correlations were positive and when the extended environment was highly positively correlated (0.84) with any of the complete phenotyping environments, sparse phenotyping was always superior (Table 1; Figure 3; Supplementary Tables 1, 2). However, this superiority was not maintained when additional environment(s) were included that were only poorly correlated with the complete phenotyping environments (Figures 4, 5; Supplementary Tables 1, 2). As there was no expansion environment with a high positive correlation (0.84) with the complete phenotyping environments in combinations 1–4, it was not possible to determine whether adding such a highly positively correlated expansion environment would be beneficial or not. It is therefore possible the efficacy of sparse phenotyping is actually very similar in groups 1 and 2.

Group 3 had two pairs of environments with a positive correlation and one pair with a negative correlation. Here, the sparse phenotyping method consistently resulted in an additional selection response when the expansion environment was highly positively correlated (0.84) or even when several expansion environments were moderately positively correlated with the complete phenotyping environments (Table 1; Figures 3, 4; Supplementary Tables 1, 2). This suggests that the robustness of group 3 is less than groups 1 and 2, and the superiority of including two expansion environments in group 3 depends on the relationship between the two expansion environments. In combination 17–18, no expansion environment was highly positively correlated with any of the complete phenotyping environments. However, two expansion environments were highly correlated (0.84), i.e., Year2015_TOS1 and Year2015_TOS3, and each was moderately positively correlated with one of the complete phenotyping environments, which made sparse phenotyping superior (Figure 4; Supplementary Table 2). In contrast, their *per se* 4-environment sparse phenotyping scenario did not show superiority (Figure 3; Supplementary Table 2).

For group 4, where one pair of environments had a positive correlation and two pairs a negative correlation, i.e., combinations 8–10, 14–16, and 20, sparse phenotyping resulted in a greater response when one expansion environment was highly correlated (0.84) or all expansion environments had moderate positive correlations with the complete phenotyping environments (Table 1; Figures 3–5; Supplementary Tables 1, 2). In some cases, such as combination 16 and 20, even one extended environment with a moderate positive correlation with the complete phenotyping environments was superior (Table 1; Figure 3). This suggests that when environments are dissimilar, the sparse phenotyping method is particularly useful; a finding corroborated by the largest number of superior 5- and 6-environment combinations in group 4 (Figures 4, 5).

The relationship between correlations of environments and the benefit of sparse phenotyping was confirmed in the rice data set even though the range of correlations between environments was not as great as that observed in wheat.

Breeders are advised to consider the expected phenotypic correlation between environments when deciding whether genomics-assisted sparse phenotyping is of value. For instance, inspecting the correlations between environments observed in the wheat data set shown in Table 1, when the environments projected for complete phenotyping contain a highly positive correlation, the sparse phenotyping method does not increase selection response. For any other combination of complete phenotyping environments, adding one expansion environment that is positively highly correlated with any of the complete phenotyping environments will always be beneficial. When most complete phenotyping environments are negatively correlated, including more (≤3) expansion environments also consistently improved the response as long as one positive highly correlated expansion environment was added. It is worth noting that while adding one highly positively correlated expansion environment was of benefit, breeders could choose this environment for complete phenotyping if some prior knowledge was available, which would revert the combination to group 1. Nevertheless, adding positive correlation sparse phenotyping scenarios was generally of benefit (group 4, Figure 3). However, in practice, breeders tend to choose environments that are distinct to select germplasm that are widely adapted.

It is also worth noting that the sparse phenotyping scenarios with less testing environments, e.g., one extended environment (4-environment combination) showed higher responses to selection than those with more environments, e.g., two and three extended environments (5- and 6-environment combinations; Figure 6; Supplementary Figure 7), which in part contradicts the experience on regular complete phenotyping that more testing environments imply higher selection accuracy and response to selection. Therefore, breeders may want to use one expansion environment when applying the sparse phenotyping approach as it would lead to a higher response. This would also facilitate the selection of extended environments as sparse phenotyping with more than one extended environment needs consideration of correlations between extended environments, which complicates the efficacy of the sparse phenotyping method.

Finally, although the budgets of the sparse phenotyping method with different number of expansion environments are theoretically identical, the actual cost would rise if the number of environments was increased, regardless of size. Hence, breeders should assess the practicality of the genomics-assisted sparse phenotyping approach based on both the relationship between testing environments and complexity of breeding program deployment.

## Conclusion

Our study demonstrated that a genomics-assisted sparse phenotyping method can improve selection response for crop breeding, especially at the middle stages of a breeding program when multi-environment trials are not feasible due to cost. The sparse phenotyping approach was optimal when most of the complete phenotyping environments were negatively or lowly positively correlated and at least one of the extension environments was positively highly correlated with any of the complete phenotyping environment.

## Data Availability Statement

The data analyzed in this study is subject to the following licenses/restrictions: wheat data is available upon request for non-commercial purposes. Requests to access these datasets should be directed to HD, hans.daetwyler@agriculture.vic.gov.au.

## Author Contributions

SH, HD, and YJ designed the study. SH conducted genomic prediction analyses and response simulations. RTr and RTh developed the plant populations and collected the phenotypes. MH oversaw genotyping. SH and HD wrote the manuscript. All authors contributed to the article and approved the submitted version.

## Funding

This study is funded by the Grain Research Development Corporation (GRDC, US00081), the University of Sydney and Agriculture Victoria.

## Conflict of Interest

The authors declare that the research was conducted in the absence of any commercial or financial relationships that could be construed as a potential conflict of interest.

## Publisher’s Note

All claims expressed in this article are solely those of the authors and do not necessarily represent those of their affiliated organizations, or those of the publisher, the editors and the reviewers. Any product that may be evaluated in this article, or claim that may be made by its manufacturer, is not guaranteed or endorsed by the publisher.
