# Supplementary material for: Improving Selection Efficiency of Crop Breeding With Genomic Prediction Aided Sparse Phenotyping
Source: Front Plant Sci. 2021 Oct 6;12:735285. doi: 10.3389/fpls.2021.735285 (PMC8526887; doi:10.3389/fpls.2021.735285)
Supplement: Supplementary file 1 [file Data_Sheet_1.docx]

**Supplementary Table 1** Wheat - Correlations between environments within all 3-environment combinations (complete phenotyping trial) and between environments in the 3-environment combinations and the expansion environments. The order of correlations within 3-environment combinations is 1^st^ and 2^nd^, 1^st^ and 3^rd^, and 2^nd^ and 3^rd^. The order of correlations between environment in the 3-environment combinations and the expansion environments corresponds to the order of environments in the 3-environment combinations

|  | Environments in the 3-enviroment combination | Correlations between environments within 3-environment combinations | Expansion Environments | Correlation between environment in the 3-environment combinations and the expansion environments |
| --- | --- | --- | --- | --- |
| Combination 1 | Year2015_TOS1  Year2015_TOS3  Year2016_TOS1 | 0.84, 0.041, 0.062 | Year2016_TOS3  Year2017_TOS1  Year2017_TOS3 | \| 0.378, 0.388, 0.167 \| \| --- \| \| -0.278, -0.355, 0.199 \| \| -0.274, -0.234, 0.21 \| |
| Combination 2 | Year2015_TOS1  Year2015_TOS3  Year2016_TOS3 | 0.84, 0.378, 0.388 | Year2016_TOS1  Year2017_TOS1  Year2017_TOS3 | \| 0.041, 0.062, 0.167 \| \| --- \| \| -0.278, -0.355, -0.266 \| \| -0.274, -0.234, -0.204 \| |
| Combination 3 | Year2015_TOS1  Year2015_TOS3  Year2017_TOS1 | 0.84, -0.278, -0.355 | Year2016_TOS1  Year2016_TOS3  Year2017_TOS3 | \| 0.041, 0.062, 0.199 \| \| --- \| \| 0.378, 0.388, -0.266 \| \| -0.274, -0.234, 0.322 \| |
| Combination 4 | Year2015_TOS1  Year2015_TOS3  Year2017_TOS3 | 0.84, -0.274, -0.234 | Year2016_TOS1  Year2016_TOS3  Year2017_TOS1 | \| 0.041, 0.062, 0.21 \| \| --- \| \| 0.378, 0.388, -0.204 \| \| -0.278, -0.355, 0.322 \| |
| Combination 5 | Year2015_TOS1  Year2016_TOS1  Year2016_TOS3 | 0.041, 0.378, 0.167 | Year2015_TOS3  Year2017_TOS1  Year2017_TOS3 | \| 0.84, 0.062, 0.388 \| \| --- \| \| -0.278, 0.199, -0.266 \| \| -0.274, 0.21, -0.204 \| |
| Combination 6 | Year2015_TOS1  Year2016_TOS1  Year2017_TOS1 | 0.041, -0.278, 0.199 | Year2015_TOS3  Year2016_TOS3  Year2017_TOS3 | \| 0.84, 0.062, -0.355 \| \| --- \| \| 0.378, 0.167, -0.266 \| \| -0.274, 0.21, 0.322 \| |
| Combination 7 | Year2015_TOS1  Year2016_TOS1  Year2017_TOS3 | 0.041, -0.274, 0.21 | Year2015_TOS3  Year2016_TOS3  Year2017_TOS1 | \| 0.84, 0.062, -0.234 \| \| --- \| \| 0.378, 0.167, -0.204 \| \| -0.278, 0.199, 0.322 \| |
| Combination 8 | Year2015_TOS1  Year2016_TOS3  Year2017_TOS1 | 0.378, -0.278, -0.266 | Year2015_TOS3  Year2016_TOS1  Year2017_TOS3 | \| 0.84, 0.388, -0.355 \| \| --- \| \| 0.041, 0.167, 0.199 \| \| -0.274, -0.204, 0.322 \| |
| Combination 9 | Year2015_TOS1  Year2016_TOS3  Year2017_TOS3 | 0.378, -0.274, -0.204 | Year2015_TOS3  Year2016_TOS1  Year2017_TOS1 | \| 0.84, 0.388, -0.234 \| \| --- \| \| 0.041, 0.167, 0.21 \| \| -0.278, -0.266, 0.322 \| |
| Combination 10 | Year2015_TOS1  Year2017_TOS1  Year2017_TOS3 | -0.278, -0.274, 0.322 | Year2015_TOS3  Year2016_TOS1  Year2016_TOS3 | \| 0.84, -0.355, -0.234 \| \| --- \| \| 0.041, 0.199, 0.21 \| \| 0.378, -0.266, -0.204 \| |
| Combination 11 | Year2015_TOS3  Year2016_TOS1  Year2016_TOS3 | 0.062, 0.388, 0.167 | Year2015_TOS1  Year2017_TOS1  Year2017_TOS3 | \| 0.84, 0.041, 0.378 \| \| --- \| \| -0.355, 0.199, -0.266 \| \| -0.234, 0.21, -0.204 \| |
| Combination 12 | Year2015_TOS3  Year2016_TOS1  Year2017_TOS1 | 0.062, -0.355, 0.199 | Year2015_TOS1  Year2016_TOS3  Year2017_TOS3 | \| 0.84, 0.041, -0.278 \| \| --- \| \| 0.388, 0.167, -0.266 \| \| -0.234, 0.21, 0.322 \| |
| Combination 13 | Year2015_TOS3  Year2016_TOS1  Year2017_TOS3 | 0.062, -0.234, 0.21 | Year2015_TOS1  Year2016_TOS3  Year2017_TOS1 | \| 0.84, 0.041, -0.274 \| \| --- \| \| 0.388, 0.167, -0.204 \| \| -0.355, 0.199, 0.322 \| |
| Combination 14 | Year2015_TOS3  Year2016_TOS3  Year2017_TOS1 | 0.388, -0.355, -0.266 | Year2015_TOS1  Year2016_TOS1  Year2017_TOS3 | \| 0.84, 0.378, -0.278 \| \| --- \| \| 0.062, 0.167, 0.199 \| \| -0.234, -0.204, 0.322 \| |
| Combination 15 | Year2015_TOS3  Year2016_TOS3  Year2017_TOS3 | 0.388, -0.234, -0.204 | Year2015_TOS1  Year2016_TOS1  Year2017_TOS1 | \| 0.84, 0.378, -0.274 \| \| --- \| \| 0.062, 0.167, 0.21 \| \| -0.355, -0.266, 0.322 \| |
| Combination 16 | Year2015_TOS3  Year2017_TOS1  Year2017_TOS3 | -0.355, -0.234, 0.322 | Year2015_TOS1  Year2016_TOS1  Year2016_TOS3 | \| 0.84, -0.278, -0.274 \| \| --- \| \| 0.062, 0.199, 0.21 \| \| 0.388, -0.266, -0.204 \| |
| Combination 17 | Year2016_TOS1  Year2016_TOS3  Year2017_TOS1 | 0.167, 0.199, -0.266 | Year2015_TOS1  Year2015_TOS3  Year2017_TOS3 | \| 0.041, 0.378, -0.278 \| \| --- \| \| 0.062, 0.388, -0.355 \| \| 0.21, -0.204, 0.322 \| |
| Combination 18 | Year2016_TOS1  Year2016_TOS3  Year2017_TOS3 | 0.167, 0.21, -0.204 | Year2015_TOS1  Year2015_TOS3  Year2017_TOS1 | \| 0.041, 0.378, -0.274 \| \| --- \| \| 0.062, 0.388, -0.234 \| \| 0.199, -0.266, 0.322 \| |
| Combination 19 | Year2016_TOS1  Year2017_TOS1  Year2017_TOS3 | 0.199, 0.21, 0.322 | Year2015_TOS1  Year2015_TOS3  Year2016_TOS3 | \| 0.041, -0.278, -0.274 \| \| --- \| \| 0.062, -0.355, -0.234 \| \| 0.167, -0.266, -0.204 \| |
| Combination 20 | Year2016_TOS3  Year2017_TOS1  Year2017_TOS3 | -0.266, -0.204, 0.322 | Year2015_TOS1  Year2015_TOS3  Year2016_TOS1 | \| 0.378, -0.278, -0.274 \| \| --- \| \| 0.388, -0.355, -0.234 \| \| 0.167, 0.199, 0.21 \| |

**Supplementary Table 2** Wheat - Student’s t-test of the difference between responses to selection of each 3-environment combination with complete phenotypic values and its extended 4-, 5- and 6-environment combinations using genomics-assisted sparse phenotyping. The asterisk indicates the expansion environment(s) resulted in statistically significant higher (P<0.05) response to selection than the 3-environment complete phenotyping trial under each selection ratio (10%-90%)

|  | **Environments in the 3-enviroment combination** | **Expansion environment establishing 4-environment combination** | **t-test** | **Expansion environment establishing 5-environment combination** | **t-test** | **Expansion environment establishing 6 environments** | **t-test** |
| --- | --- | --- | --- | --- | --- | --- | --- |
| Combination 1 | Year2015_TOS1,  Year2015_TOS3,  Year2016_TOS1 | Year2016_TOS3 |  | Year2016_TOS3,  Year2017_TOS1 |  | Year2016_TOS3,  Year2017_TOS1,  Year2017_TOS3 |  |
|  |  | Year2017_TOS1 |  | Year2016_TOS3,  Year2017_TOS3 |  |  |  |
|  |  | Year2017_TOS3 |  | Year2017_TOS1,  Year2017_TOS3 |  |  |  |
| Combination 2 | Year2015_TOS1,  Year2015_TOS3,  Year2016_TOS3 | Year2016_TOS1 |  | Year2016_TOS1,  Year2017_TOS1 |  | Year2016_TOS1,  Year2017_TOS1,  Year2017_TOS3 |  |
|  |  | Year2017_TOS1 |  | Year2016_TOS1,  Year2017_TOS3 |  |  |  |
|  |  | Year2017_TOS3 |  | Year2017_TOS1,  Year2017_TOS3 |  |  |  |
| Combination 3 | Year2015_TOS1,  Year2015_TOS3,  Year2017_TOS1 | Year2016_TOS1 |  | Year2016_TOS1,  Year2016_TOS3 |  | Year2016_TOS1,  Year2016_TOS3,  Year2017_TOS3 |  |
|  |  | Year2016_TOS3 |  | Year2016_TOS1,  Year2017_TOS3 |  |  |  |
|  |  | Year2017_TOS3 |  | Year2016_TOS3,  Year2017_TOS3 |  |  |  |
| Combination 4 | Year2015_TOS1,  Year2015_TOS3,  Year2017_TOS3 | Year2016_TOS1 |  | Year2016_TOS1,  Year2016_TOS3 |  | Year2016_TOS1,  Year2016_TOS3,  Year2017_TOS1 |  |
|  |  | Year2016_TOS3 |  | Year2016_TOS1,  Year2017_TOS1 |  |  |  |
|  |  | Year2017_TOS1 |  | Year2016_TOS3,  Year2017_TOS1 |  |  |  |
| Combination 5 | Year2015_TOS1,  Year2016_TOS1,  Year2016_TOS3 | Year2015_TOS3 | * | Year2015_TOS3,  Year2017_TOS1 |  | Year2015_TOS3,  Year2017_TOS1,  Year2017_TOS3 |  |
|  |  | Year2017_TOS1 |  | Year2015_TOS3,  Year2017_TOS3 |  |  |  |
|  |  | Year2017_TOS3 |  | Year2017_TOS1,  Year2017_TOS3 |  |  |  |
| Combination 6 | Year2015_TOS1,  Year2016_TOS1,  Year2017_TOS1 | Year2015_TOS3 | * | Year2015_TOS3,  Year2016_TOS3 | * | Year2015_TOS3,  Year2016_TOS3,  Year2017_TOS3 |  |
|  |  | Year2016_TOS3 |  | Year2015_TOS3,  Year2017_TOS3 |  |  |  |
|  |  | Year2017_TOS3 |  | Year2016_TOS3,  Year2017_TOS3 |  |  |  |
| Combination 7 | Year2015_TOS1,  Year2016_TOS1,  Year2017_TOS3 | Year2015_TOS3 | * | Year2015_TOS3,  Year2016_TOS3 | * | Year2015_TOS3,  Year2016_TOS3,  Year2017_TOS1 |  |
|  |  | Year2016_TOS3 |  | Year2015_TOS3,  Year2017_TOS1 |  |  |  |
|  |  | Year2017_TOS1 |  | Year2016_TOS3,  Year2017_TOS1 |  |  |  |
| Combination 8 | Year2015_TOS1,  Year2016_TOS3,  Year2017_TOS1 | Year2015_TOS3 | * | Year2015_TOS3,  Year2016_TOS1 | * | Year2015_TOS3,  Year2016_TOS1,  Year2017_TOS3 | * |
|  |  | Year2016_TOS1 | * | Year2015_TOS3,  Year2017_TOS3 | * |  |  |
|  |  | Year2017_TOS3 |  | Year2016_TOS1,  Year2017_TOS3 |  |  |  |
| Combination 9 | Year2015_TOS1,  Year2016_TOS3,  Year2017_TOS3 | Year2015_TOS3 | * | Year2015_TOS3,  Year2016_TOS1 | * | Year2015_TOS3,  Year2016_TOS1,  Year2017_TOS1 | * |
|  |  | Year2016_TOS1 | * | Year2015_TOS3,  Year2017_TOS1 | * |  |  |
|  |  | Year2017_TOS1 |  | Year2016_TOS1,  Year2017_TOS1 |  |  |  |
| Combination 10 | Year2015_TOS1,  Year2017_TOS1,  Year2017_TOS3 | Year2015_TOS3 | * | Year2015_TOS3,  Year2016_TOS1 | * | Year2015_TOS3,  Year2016_TOS1,  Year2016_TOS3 | * |
|  |  | Year2016_TOS1 | * | Year2015_TOS3,  Year2016_TOS3 | * |  |  |
|  |  | Year2016_TOS3 |  | Year2016_TOS1,  Year2016_TOS3 | * |  |  |
| Combination 11 | Year2015_TOS3,  Year2016_TOS1,  Year2016_TOS3 | Year2015_TOS1 | * | Year2015_TOS1,  Year2017_TOS1 |  | Year2015_TOS1,  Year2017_TOS1,  Year2017_TOS3 |  |
|  |  | Year2017_TOS1 |  | Year2015_TOS1,  Year2017_TOS3 |  |  |  |
|  |  | Year2017_TOS3 |  | Year2017_TOS1,  Year2017_TOS3 |  |  |  |
| Combination 12 | Year2015_TOS3,  Year2016_TOS1,  Year2017_TOS1 | Year2015_TOS1 | * | Year2015_TOS1,  Year2016_TOS3 | * | Year2015_TOS1,  Year2016_TOS3,  Year2017_TOS3 |  |
|  |  | Year2016_TOS3 |  | Year2015_TOS1,  Year2017_TOS3 |  |  |  |
|  |  | Year2017_TOS3 |  | Year2016_TOS3,  Year2017_TOS3 |  |  |  |
| Combination 13 | Year2015_TOS3,  Year2016_TOS1,  Year2017_TOS3 | Year2015_TOS1 | * | Year2015_TOS1,  Year2016_TOS3 | * | Year2015_TOS1,  Year2016_TOS3,  Year2017_TOS1 |  |
|  |  | Year2016_TOS3 |  | Year2015_TOS1,  Year2017_TOS1 |  |  |  |
|  |  | Year2017_TOS1 |  | Year2016_TOS3,  Year2017_TOS1 |  |  |  |
| Combination 14 | Year2015_TOS3,  Year2016_TOS3,  Year2017_TOS1 | Year2015_TOS1 | * | Year2015_TOS1,  Year2016_TOS1 | * | Year2015_TOS1,  Year2016_TOS1,  Year2017_TOS3 | * |
|  |  | Year2016_TOS1 | * | Year2015_TOS1,  Year2017_TOS3 | * |  |  |
|  |  | Year2017_TOS3 |  | Year2016_TOS1,  Year2017_TOS3 |  |  |  |
| Combination 15 | Year2015_TOS3,  Year2016_TOS3,  Year2017_TOS3 | Year2015_TOS1 | * | Year2015_TOS1,  Year2016_TOS1 | * | Year2015_TOS1,  Year2016_TOS1,  Year2017_TOS1 | * |
|  |  | Year2016_TOS1 | * | Year2015_TOS1,  Year2017_TOS1 | * |  |  |
|  |  | Year2017_TOS1 |  | Year2016_TOS1,  Year2017_TOS1 |  |  |  |
| Combination 16 | Year2015_TOS3,  Year2017_TOS1,  Year2017_TOS3 | Year2015_TOS1 | * | Year2015_TOS1,  Year2016_TOS1 | * | Year2015_TOS1,  Year2016_TOS1,  Year2016_TOS3 | * |
|  |  | Year2016_TOS1 | * | Year2015_TOS1,  Year2016_TOS3 | * |  |  |
|  |  | Year2016_TOS3 | * | Year2016_TOS1,  Year2016_TOS3 | * |  |  |
| Combination 17 | Year2016_TOS1,  Year2016_TOS3,  Year2017_TOS1 | Year2015_TOS1 |  | Year2015_TOS1,  Year2015_TOS3 | * | Year2015_TOS1,  Year2015_TOS3,  Year2017_TOS3 |  |
|  |  | Year2015_TOS3 |  | Year2015_TOS1,  Year2017_TOS3 |  |  |  |
|  |  | Year2017_TOS3 |  | Year2015_TOS3,  Year2017_TOS3 |  |  |  |
| Combination 18 | Year2016_TOS1,  Year2016_TOS3,  Year2017_TOS3 | Year2015_TOS1 |  | Year2015_TOS1,  Year2015_TOS3 | * | Year2015_TOS1,  Year2015_TOS3,  Year2017_TOS1 |  |
|  |  | Year2015_TOS3 |  | Year2015_TOS1,  Year2017_TOS1 |  |  |  |
|  |  | Year2017_TOS1 |  | Year2015_TOS3,  Year2017_TOS1 |  |  |  |
| Combination 19 | Year2016_TOS1,  Year2017_TOS1,  Year2017_TOS3 | Year2015_TOS1 |  | Year2015_TOS1,  Year2015_TOS3 |  | Year2015_TOS1,  Year2015_TOS3,  Year2016_TOS3 |  |
|  |  | Year2015_TOS3 |  | Year2015_TOS1,  Year2016_TOS3 |  |  |  |
|  |  | Year2016_TOS3 |  | Year2015_TOS3,  Year2016_TOS3 |  |  |  |
| Combination 20 | Year2016_TOS3,  Year2017_TOS1,  Year2017_TOS3 | Year2015_TOS1 | * | Year2015_TOS1,  Year2015_TOS3 | * | Year2015_TOS1,  Year2015_TOS3,  Year2016_TOS1 | * |
|  |  | Year2015_TOS3 |  | Year2015_TOS1,  Year2016_TOS1 | * |  |  |
|  |  | Year2016_TOS1 | * | Year2015_TOS3,  Year2016_TOS1 | * |  |  |

**
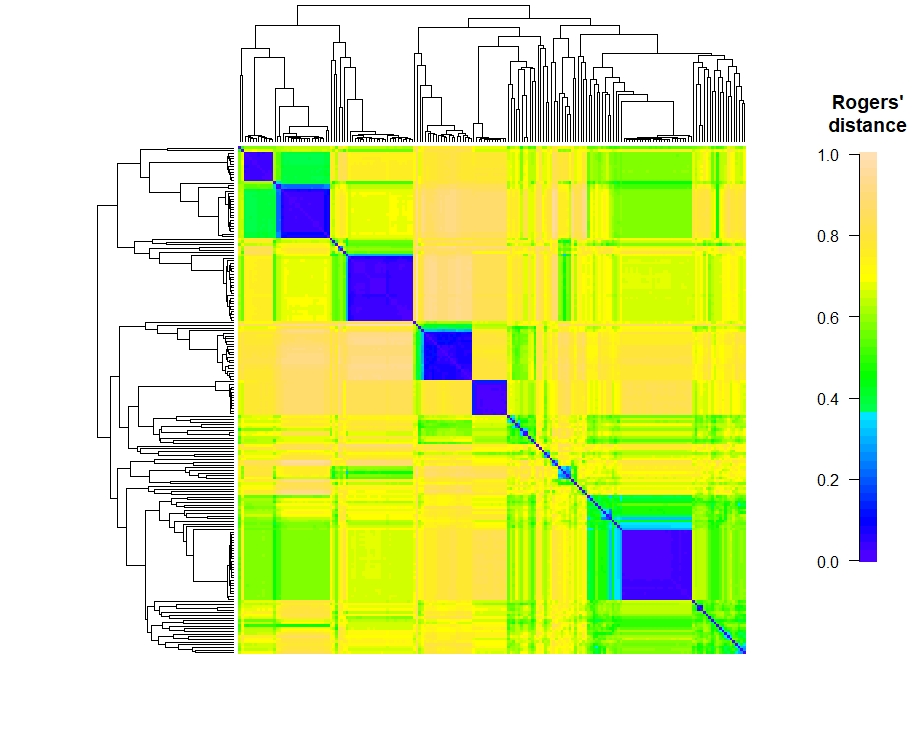
**

**Supplementary Figure 1** Wheat - Pairwise genetic dissimilarities among 189 wheat genotypes described by Rogers’ distance. The complete clustering method was used to order the genotypes


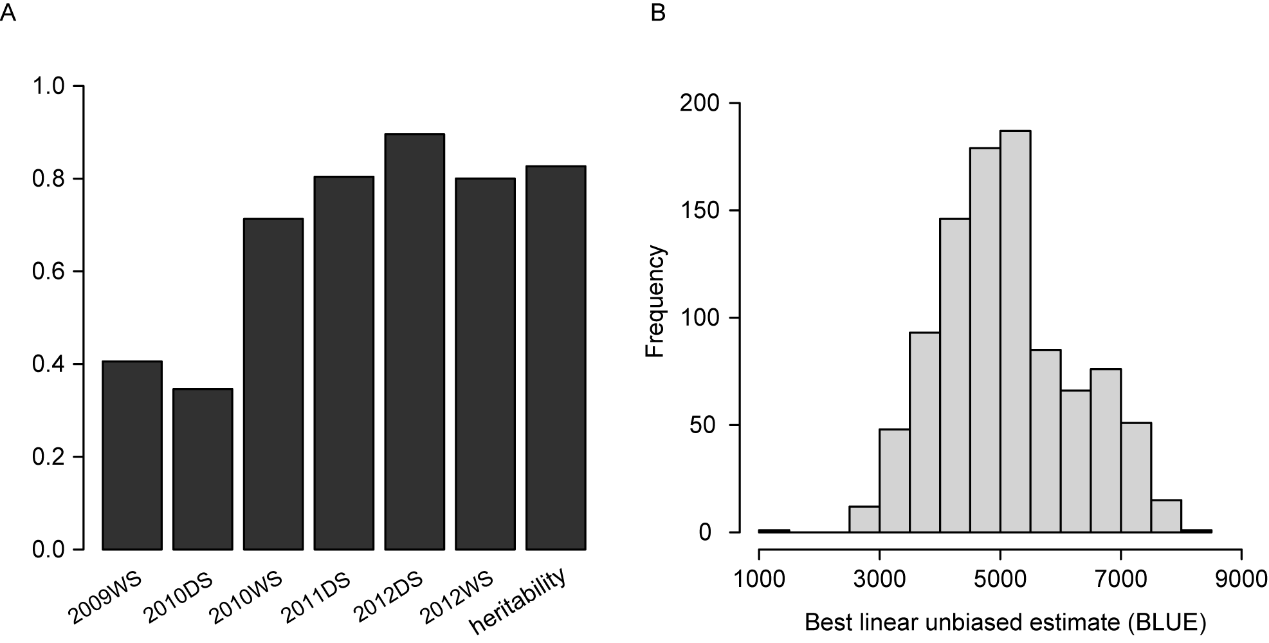


**Supplementary Figure 2** Rice - (A) Heritability of grain yield and repeatability in each environment; (B) Distribution of best linear unbiased estimate (BLUE) of genotypes in different environments


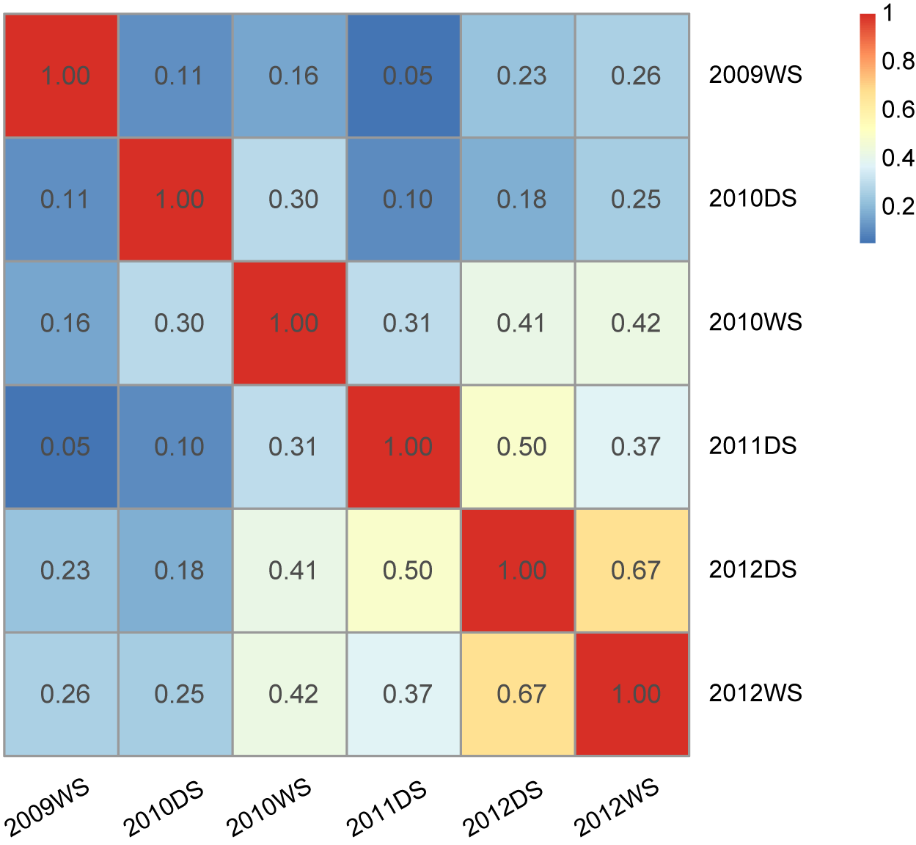


**Supplementary Figure 3** Rice - Pairwise correlation between environments


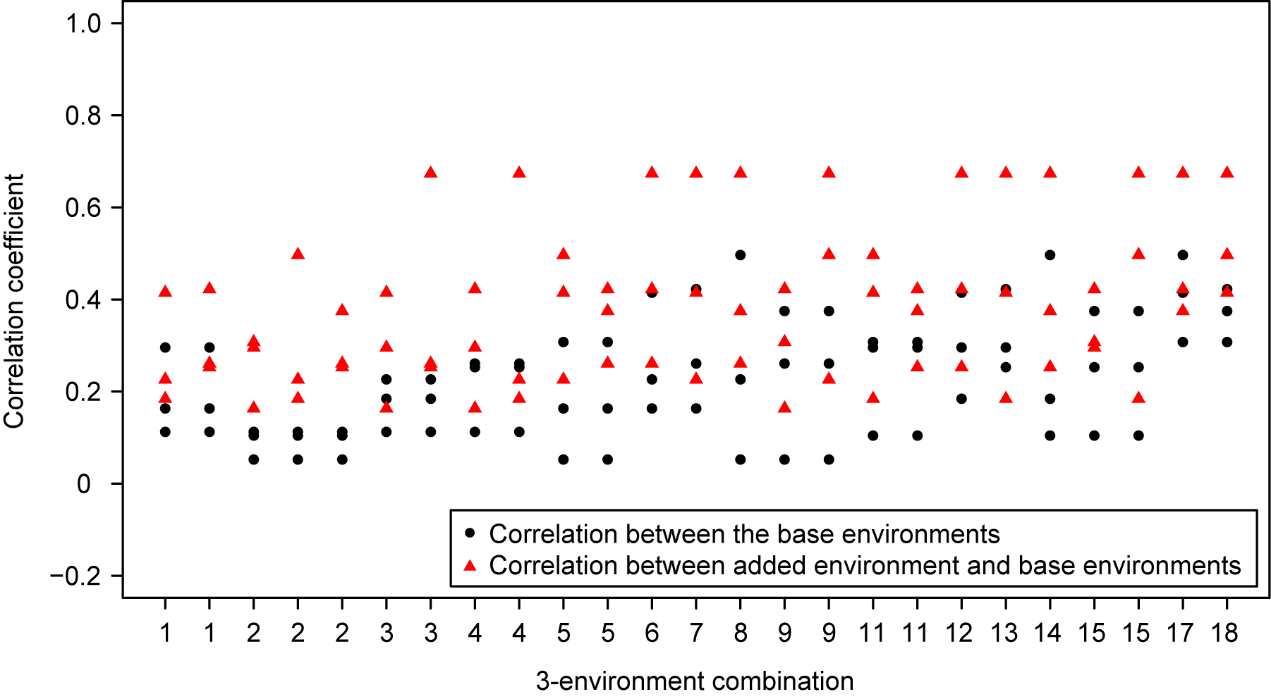


**Supplementary Figure 4** Rice - 3-environment combinations with complete phenotypic values showing statistically significant (P<0.05) lower response to selection than their extended 4-environment combinations using genomics-assisted sparse phenotyping. Labels of horizontal axis are the scenario numbers of 3-environment combinations. Black dots represent correlation coefficients between the three base environments with complete phenotypic values. Red triangles indicate correlation coefficients between the added environment and base environments


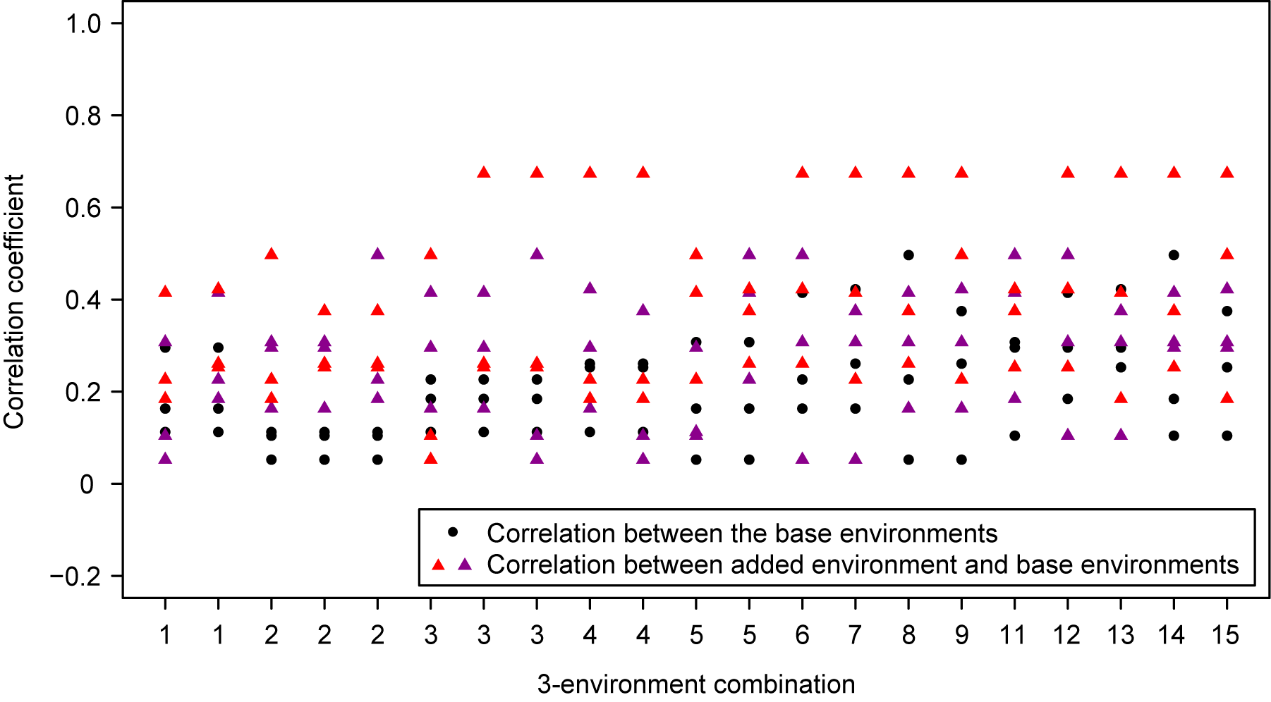


**Supplementary Figure 5** Rice - 3-environment combinations with complete phenotypic values showing statistically significant (P<0.05) lower response to selection than their extended 5-environment combinations using genomics-assisted sparse phenotyping. Labels of horizontal axis are the scenario numbers of 3-environment combinations. Black dots represent correlation coefficients between the three base environments with complete phenotypic values. Triangles with different colors indicate correlation coefficients between separate added environments, i.e., the first or second added environment, and base environments


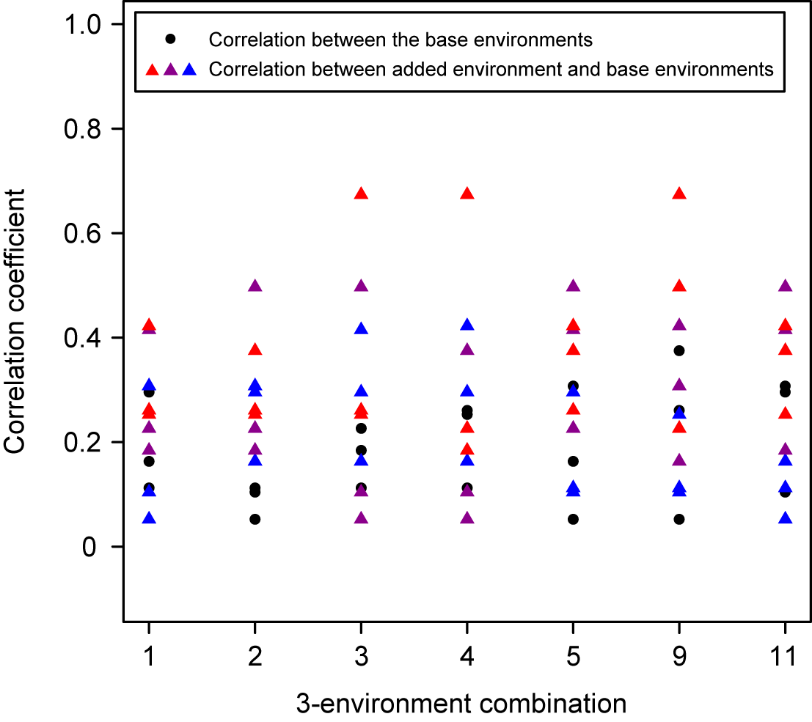


**Supplementary Figure 6** Rice - 3-environment combinations with complete phenotypic values showing statistically significant (P<0.05) lower response to selection than using total six environments with genomics-assisted sparse phenotyping. Labels of horizontal axis are the scenario numbers of 3-environment combinations. Black dots represent correlation coefficients between the three base environments with complete phenotypic values. Triangles with different colors indicate correlation coefficients between separate added environments, i.e., the first, second or third added environment, and base environments


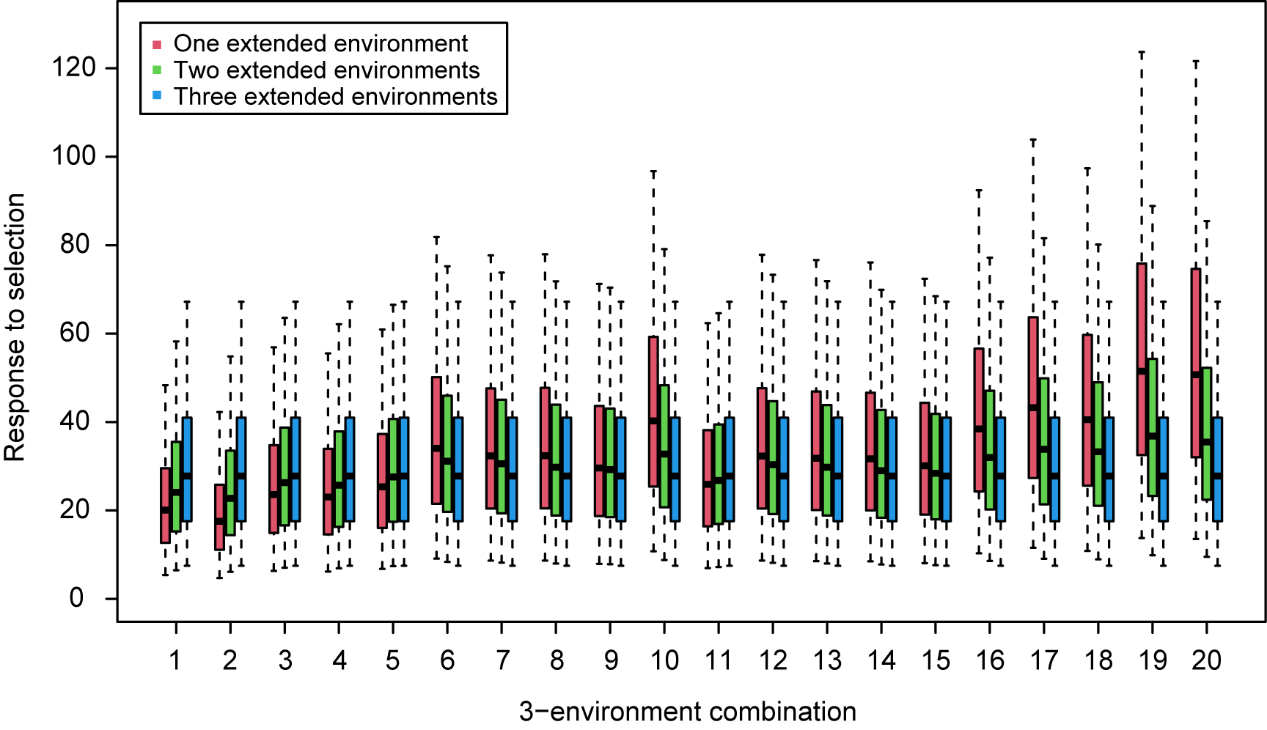


**Supplementary Figure 7** Rice - responses to selection of 4-environment (one extended environment), 5-environment (two extended environments) and 6-environment (three extended environments) sparse phenotyping combinations belonging to each 3-environment complete phenotyping combination. Labels of horizontal axis are the scenario numbers of 3-environment combinations


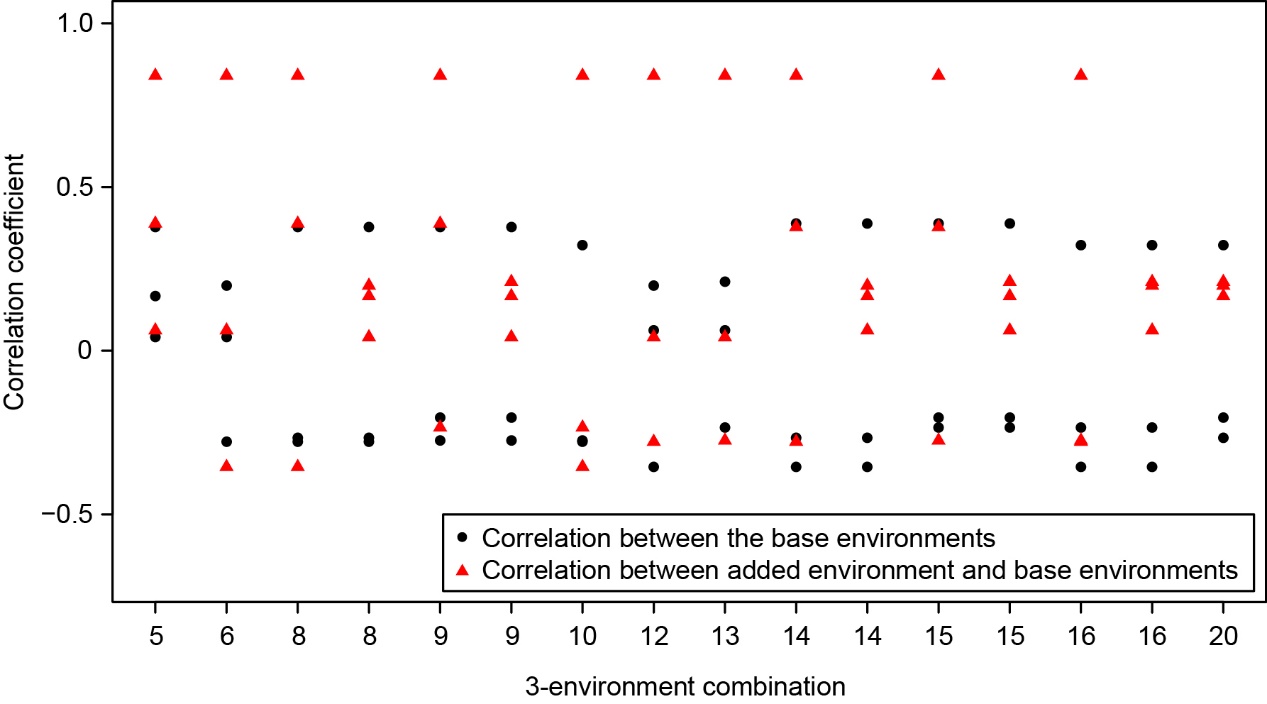


**Supplementary Figure 8** Wheat - 3-environment combinations with complete phenotypic values showing statistically significant (P<0.05) lower response to selection than their extended 4-environment combinations using genomics-assisted sparse phenotyping. Relationship between environments was considered in multi-environment genomic prediction model and simulation of response to selection. Environmental relationship matrix was estimated using pairwise complete observations (BLUEs) between environments. Labels of horizontal axis are the scenario numbers of 3-environment combinations. Black dots represent correlation coefficients between the three base environments with complete phenotypic values. Red triangles indicate correlation coefficients between the added environment and base environments


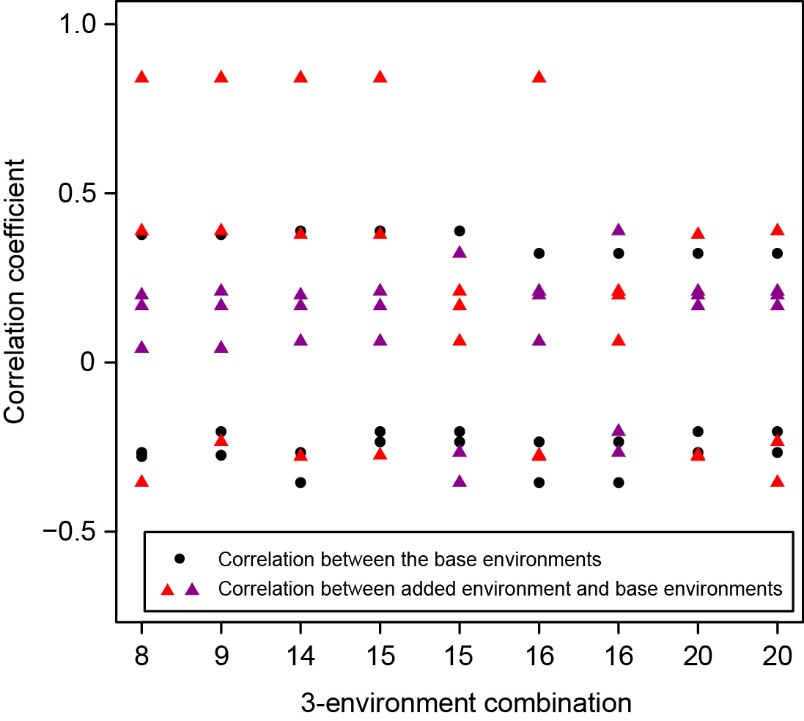


**Supplementary Figure 9** Wheat - 3-environment combinations with complete phenotypic values showing statistically significant (P<0.05) lower response to selection than their extended 5-environment combinations using genomics-assisted sparse phenotyping. Relationship between environments was considered in multi-environment genomic prediction model and simulation of response to selection. Environmental relationship matrix was estimated using pairwise complete observations (BLUEs) between environments. Labels of horizontal axis are the scenario numbers of 3-environment combinations. Black dots represent correlation coefficients between the three base environments with complete phenotypic values. Triangles with different colors indicate correlation coefficients between separate added environments, i.e., the first or second added environment, and base environments


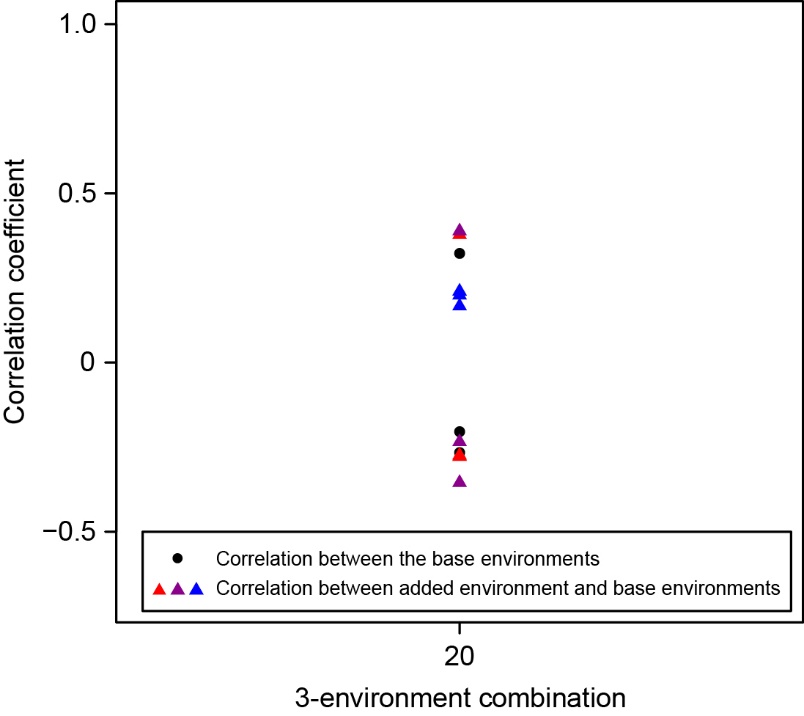


**Supplementary Figure 10** Wheat - 3-environment combinations with complete phenotypic values showing statistically significant (P<0.05) lower response to selection than using total six environments with genomics-assisted sparse phenotyping. Relationship between environments was considered in multi-environment genomic prediction model and simulation of response to selection. Environmental relationship matrix was estimated using pairwise complete observations (BLUEs) between environments. Label of horizontal axis is the scenario number of 3-environment combinations. Black dots represent correlation coefficients between the three base environments with complete phenotypic values. Triangles with different colors indicate correlation coefficients between separate added environment, i.e., the first, second or third added environment, and base environments


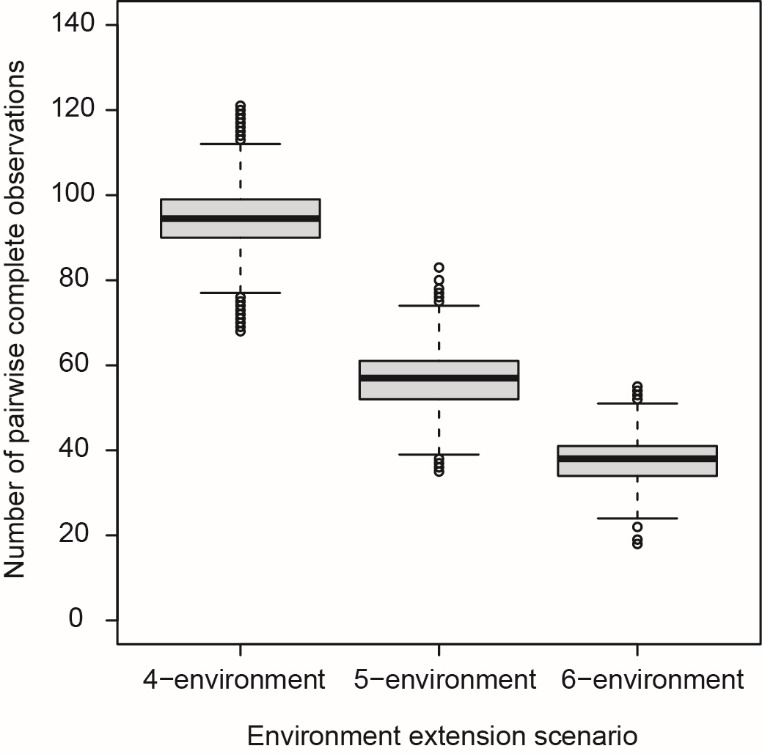


**Supplementary Figure 11** Wheat - Number of pairwise complete observations (BLUEs) used for estimating environmental relationship matrix in multi-environment genomic prediction model under different environment extension scenarios.

########################################

### Script of simulating response to selection ###

########################################

rm(list=ls())

library(BGLR) # load BGLR package

Nr_envs <- 4 # Number of environments in sparse phenotyping: 4 means one expansion environment

Nr_rep <- 100 # Number of replications of simulating sparse phenotyping pattern

GRM_ori <- as.matrix(read.table('kinship.txt',as.is = T)) # genotypic relationship matrix

combn_Envs_all <- combn(6,Nr_envs) # generate combinations

for (column in 1:ncol(combn_Envs_all))

{

Res2Sel_all <- c()

Y <- read.table(paste0('combn',column,'/Y.txt'),header = T,as.is = T) # phenotypic data file (3 columns): 1st column is BLUE, 2nd is ID, 3rd is Environment

ones <- rep(1,nrow(Y))

X <- as.matrix(ones)

colnames(X) <- 'intercept'

Envs <- unique(Y$Environment)

nEnv <- length(Envs)

Envs.multiEnv <- Y$Environment

Envs.multiEnv <- factor(Envs.multiEnv,levels=Envs)

Zenv <- as.matrix(model.matrix(~Envs.multiEnv-1))

G.E <- tcrossprod(Zenv)

EVD.E <- eigen(G.E)

IDs <- unique(Y$ID)

nID <- length(IDs)

IDs.multiEnv <- Y$ID

IDs.multiEnv <- factor(IDs.multiEnv,levels=IDs)

Zgeno <- as.matrix(model.matrix(~IDs.multiEnv-1))

GRM <- GRM_ori[IDs,IDs]

EVD <- eigen(GRM)

eigval <- EVD$values

eigvec <- EVD$vectors

eigval[eigval<1e-6] <- 1e-6

GRM <- eigvec %*% diag(eigval) %*% t(eigvec)

GRM_inv <- eigvec %*% diag(1/eigval) %*% t(eigvec)

rownames(GRM) <- IDs

colnames(GRM) <- IDs

rownames(GRM_inv) <- IDs

colnames(GRM_inv) <- IDs

G.G <- tcrossprod(tcrossprod(Zgeno,GRM),Zgeno)

EVD.G<-eigen(G.G)

G.GxE <- G.G*G.E

EVD.GxE <- eigen(G.GxE)

ID.Env.Int <- paste(Y$ID,Y$Environment,sep = ':')

G.GxE_inv <- solve(G.GxE)

rownames(G.GxE_inv) <- ID.Env.Int

colnames(G.GxE_inv) <- ID.Env.Int

Zint <- diag(1:length(ID.Env.Int))

K_E<-list(V=EVD.E$vectors,d=EVD.E$values,model="RKHS")

K_G<-list(V=EVD.G$vectors,d=EVD.G$values,model="RKHS")

K_GxE<-list(V=EVD.GxE$vectors,d=EVD.GxE$values,model="RKHS")

ETA <- list(K_E,K_G,K_GxE)

for (jj in 1:Nr_rep)

{

TestData <- scan(paste0('combn',column,'/sampling/TestData_',jj,'.txt')) # one sample of sparse phenotyping pattern

yNA <- Y$BLUE

yNA[TestData] <- NA

fm <- BGLR(y=yNA,

ETA=ETA,

nIter=30000,

burnIn=5000,

saveAt=paste0("combn",column,"/BGLR/BGLR_",paste(Envs,collapse = "_"),"_rep",jj,""),

verbose=FALSE)

varcomp.env <- fm$ETA[[1]]$varU

varcomp.gen <- fm$ETA[[2]]$varU

varcomp.gxE <- fm$ETA[[3]]$varU

varcomp.err <- fm$varE

Y_shrink <- Y[!is.na(yNA),]

ones_shrink <- rep(1,nrow(Y_shrink))

X_shrink <- as.matrix(ones_shrink)

colnames(X_shrink) <- 'intercept'

Envs_shrink <- unique(Y_shrink$Environment)

nEnv_shrink <- length(Envs_shrink)

Envs_shrink.multiEnv <- Y_shrink$Environment

Envs_shrink.multiEnv <- factor(Envs_shrink.multiEnv,levels=Envs_shrink)

Zenv_shrink <- as.matrix(model.matrix(~Envs_shrink.multiEnv-1))

G.E_shrink <- tcrossprod(Zenv_shrink)

IDs_shrink <- unique(Y_shrink$ID)

nID_shrink <- length(IDs_shrink)

IDs_shrink.multiEnv <- Y_shrink$ID

IDs_shrink.multiEnv <- factor(IDs_shrink.multiEnv,levels=IDs_shrink)

Zgeno_shrink <- as.matrix(model.matrix(~IDs_shrink.multiEnv-1))

GRM_shrink <- GRM_ori[IDs_shrink,IDs_shrink]

EVD_shrink <- eigen(GRM_shrink)

eigval_shrink <- EVD_shrink$values

eigvec_shrink <- EVD_shrink$vectors

eigval_shrink[eigval_shrink<1e-6] <- 1e-6

GRM_shrink <- eigvec_shrink %*% diag(eigval_shrink) %*% t(eigvec_shrink)

GRM_inv_shrink <- eigvec_shrink %*% diag(1/eigval_shrink) %*% t(eigvec_shrink)

rownames(GRM_shrink) <- IDs_shrink

colnames(GRM_shrink) <- IDs_shrink

rownames(GRM_inv_shrink) <- IDs_shrink

colnames(GRM_inv_shrink) <- IDs_shrink

G.G_shrink <- tcrossprod(tcrossprod(Zgeno_shrink,GRM_shrink),Zgeno_shrink)

G.GxE_shrink <- G.G_shrink*G.E_shrink

EVD.GxE_shrink <- eigen(G.GxE_shrink)

ID.Env.Int_shrink <- paste(Y_shrink$ID,Y_shrink$Environment,sep = ':')

G.GxE_inv_shrink <- solve(G.GxE_shrink)

rownames(G.GxE_inv_shrink) <- ID.Env.Int_shrink

colnames(G.GxE_inv_shrink) <- ID.Env.Int_shrink

Zint_shrink <- diag(1:length(ID.Env.Int_shrink))

Zcombine_shrink <- cbind(Zenv_shrink,Zgeno_shrink,Zint_shrink)

ZRZ <- crossprod(Zcombine_shrink)/varcomp.err

Gcombine <- diag(0,nEnv_shrink+nID_shrink+length(ID.Env.Int_shrink))

Gcombine[1:nEnv_shrink,1:nEnv_shrink] <- diag(1,nEnv_shrink)*varcomp.env

Gcombine[(nEnv_shrink+1):(nEnv_shrink+nID_shrink),(nEnv_shrink+1):(nEnv_shrink+nID_shrink)] <- GRM_shrink*varcomp.gen

Gcombine[(nEnv_shrink+nID_shrink+1):nrow(Gcombine),(nEnv_shrink+nID_shrink+1):ncol(Gcombine)] <- G.GxE_shrink*varcomp.gxE

Gcombine_inv <- solve(Gcombine)

leftMat1 <- crossprod(X_shrink)/varcomp.err

leftMat2 <- crossprod(X_shrink,Zcombine_shrink)/varcomp.err

leftMat3 <- crossprod(Zcombine_shrink,X_shrink)/varcomp.err

leftMat4 <- ZRZ + Gcombine_inv

leftMat <- rbind(cbind(leftMat1,leftMat2),

cbind(leftMat3,leftMat4))

leftMat_inv <- solve(leftMat)

C22 <- leftMat_inv[(nEnv_shrink+2):(nEnv_shrink+nID_shrink+1),(nEnv_shrink+2):(nEnv_shrink+nID_shrink+1)]

G <- GRM_shrink*varcomp.gen

Var.gcup<- G - C22

omegaMat <- rbind(cbind(G,Var.gcup),cbind(Var.gcup,Var.gcup))

EVD <- eigen(omegaMat)

if (all(EVD$values > 0))

{

taoMat <- EVD$vectors %*% sqrt(diag(EVD$values))

} else {

eigval <- EVD$values

eigval[eigval<1e-6] <- 1e-6

taoMat <- EVD$vectors %*% sqrt(diag(eigval))

}

Res2Sel <- c()

for (mal in 1:10000)

{

set.seed(12345+mal)

z <- rnorm(nrow(taoMat),mean = 0,sd = 1)

w <- taoMat %*% as.matrix(z)

g <- w[1:length(IDs_shrink)]

gcup <- w[(length(IDs_shrink)+1):length(w)]

names(g) <- IDs_shrink

names(gcup) <- IDs_shrink

gcup_sort <- sort(gcup,decreasing = T)

Rq_mal <- c()

for (perc in seq(0.1,0.9,0.1))

{

fraction <- round(nID_shrink*perc)

gcup_subset <- gcup_sort[1:fraction]

selected_ones <- names(gcup_subset)

g_subset <- g[selected_ones]

Rq <- sum(g_subset)/fraction

Rq_mal <- c(Rq_mal,Rq)

}

Res2Sel <- rbind(Res2Sel,Rq_mal)

}

Res2Sel <- apply(Res2Sel,2,mean)

Res2Sel_all <- rbind(Res2Sel_all,Res2Sel)

}

colnames(Res2Sel_all) <- paste0('Perc_',seq(0.1,0.9,0.1))

write.table(Res2Sel_all,file = paste0('combn',column,'/Res2Sel_EG_GxE_directEsti.txt'),quote = F,row.names = F,sep = '\t') # simulated response to selection

cat ('column:',column,' done\n')

}
